# Status of research on the development and regeneration of hair follicles

**DOI:** 10.7150/ijms.88508

**Published:** 2024-01-01

**Authors:** Dan Liu, Qi Xu, Xiangyuan Meng, Xiaomei Liu, Jinyu Liu

**Affiliations:** Department of Toxicology, School of Public Health, Jilin University, Changchun 130021, China.

**Keywords:** hair loss, hair follicle regeneration, hair follicle growth, stem cell therapy

## Abstract

Hair loss, or alopecia, is a prevalent condition in modern society that imposes substantial mental and psychological burden on individuals. The types of hair loss, include androgenetic alopecia, alopecia areata, and telogen effluvium; of them, androgenetic alopecia is the most common condition. Traditional treatment modalities mainly involve medical options, such as minoxidil, finasteride and surgical interventions, such as hair transplantation. However, these treatments still have many limitations. Therefore, exploring the pathogenesis of hair loss, specifically focusing on the development and regeneration of hair follicles (HFs), and developing new strategies for promoting hair regrowth are essential. Some emerging therapies for hair loss have gained prominence; these therapies include low-level laser therapy, micro needling, fractional radio frequency, platelet-rich plasma, and stem cell therapy. The aforementioned therapeutic strategies appear promising for hair loss management. In this review, we investigated the mechanisms underlying HF development and regeneration. For this, we studied the structure, development, cycle, and cellular function of HFs. In addition, we analyzed the symptoms, types, and causes of hair loss as well as its current conventional treatments. Our study provides an overview of the most effective regenerative medicine-based therapies for hair loss.

## 1. Introduction

With the rapid increase in social demands and work-life pressure, hair loss has become a common and increasingly severe problem. Various factors, such as trauma, mental stimulation, genetics, endocrine imbalance, physical stress, and chemical exposure, can lead to severe hair loss, affecting patients' appearance and mental health 1. Currently, the first-line treatment for hair loss involves the use of minoxidil and finasteride. However, these two drugs require long-term usage, which may result in drug tolerance and other side effects, Moreover, a hair loss often recurs upon discontinuation the drugs 2. Although hair follicle (HF) transplantation has been gradually gaining acceptance, the low availability of HF donor sites for limits patient satisfaction. With the advancement of regenerative medicine, various treatment modalities have been introduced safely and effectively resolve the problem of hair loss. These modalities include low-level laser therapy (LLLT), micro needling, platelet-rich plasma (PRP) therapy, and stem cell therapy. The modern treatments have opened up new avenues for addressing challenges associated with traditional hair loss treatment 3. In this review, we analyzed the types and causes of hair loss by exploring the molecular mechanisms underlying the development and regeneration of HFs, Herein, we discuss new treatment strategies for enhancing hair loss treatments. Our review provides a theoretical basis for the future application of stem cell therapy for hair loss.

## 2. Histomorphology of the hair follicle

**Figure 1:**
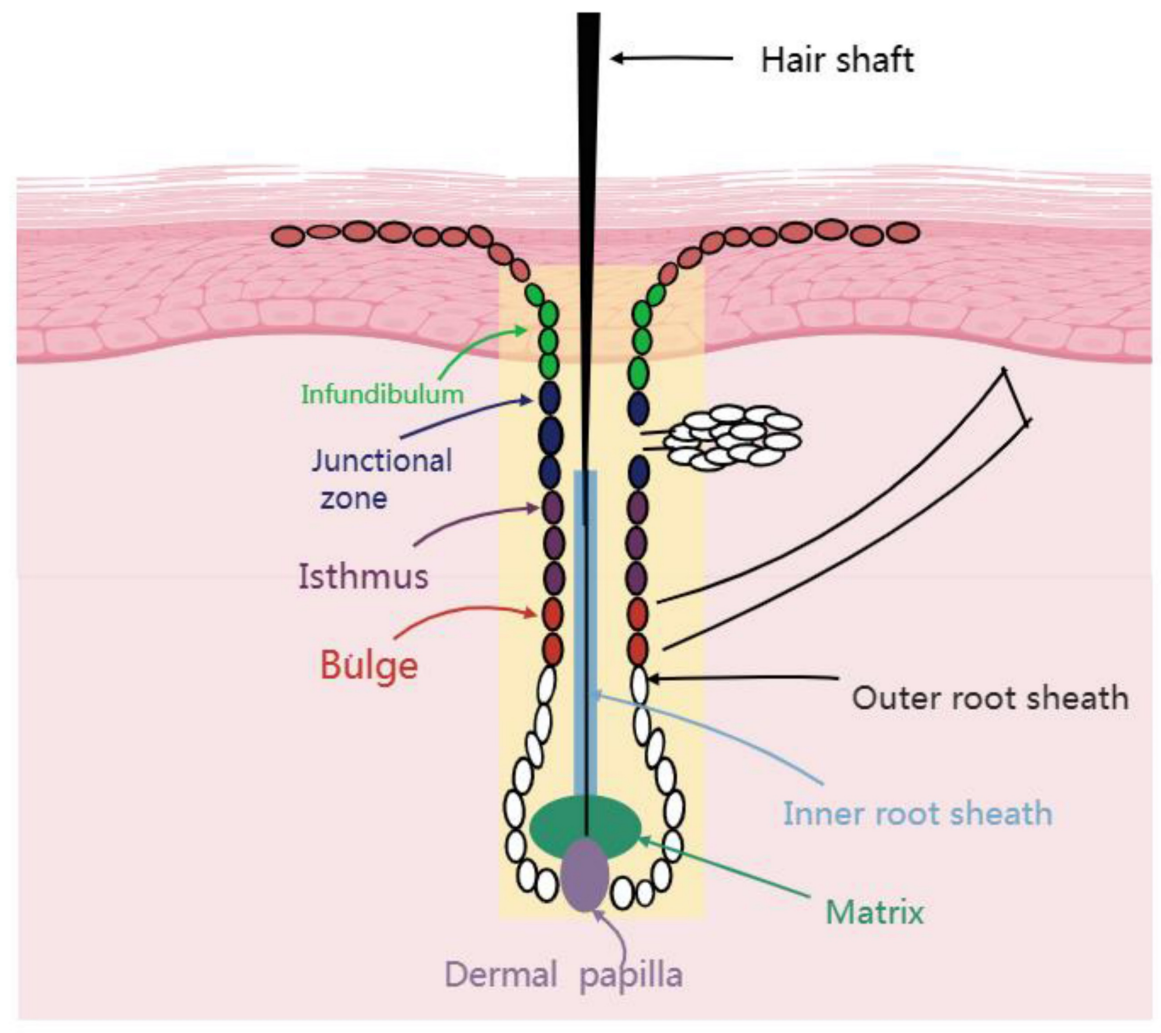
Structure of a hair follicle.

One distinctive traits of mammals are the presence of HFs, which are small skin organs. 4 Hair growth is driven by cellular activity within HFs which serve as the fundamental unit of hair 5. Although different mammalian HF exhibit different morphological forms, they have similar structural features Figure 1. The HF is divided into two portions by a boundary known as the bulge. The upper portion of the HF include the infundibulum and isthmus. The lower portion of the HF consists of the bulb, including protrusions, hairballs, and papillae. Notably, the lower portion constitutes approximately one-third of the HF and participates in the hair growth hair loss cycle. The upper portion does not participate in the cycle, because it undergoes minimal apoptosis and regeneration in the follicular phase. Thus, the upper portion is known as the permanent component, whereas the lower portion is called the cycling component 6. The layers of the HF, extending from the outside to the inside, include the outer hair root sheath (ORS), companion layer, inner hair root sheath (IRS) , and hair shaft (HS) 7, 8. HF structures, such as the IRS and HS are formed by hair matrix cells which wrap around the HFs in the body to create the hair dermal papilla (DP). The infundibulum, isthmus, bulge, and bulb of the HF are part of the HF epidermis and originate from the ectoderm. The DP a raised structure formed by the mesoderm-derived dermal tissue connected to the hairball determines the hairball size, HS diameter and length, and hair growth duration. HS is the center of the HF epidermis the entire epidermis is surrounded by a connective tissue sheath of mesodermal origin.

## 3. HF morphogenesis and development

The morphogenesis and development of HFs are dependent on epidermis-interstitial interactions. This process is regulated by various signaling pathways, particularly the synergistic actions of the Wnt, bone morphogenetic protein (BMP), hedgehog, transforming growth factor (TGF)-β, fibroblast growth factor (FGF), and Notch signaling pathways 9, 10. The Wnt/β-catenin signaling pathway is considered to be pivotal for HFs from the telogen phase to the anagen phase. This pathway is involved in all the stages of HF development and determines the differentiation fate of HF cells during development 11. BMP is a crucial member of the TGF-β superfamily. The expression levels of BMP-2 and BMP-4 changes periodically during the HF cycle 12. The Notch signaling pathway is essential for maintaining follicle structure and facilitating follicle formation and reepithelialization 13. Ultimately, determining the morphology and development of the HFs 14, 15. The morphogenesis and development of HFs proceed through three sequential stages: induction (production of hair placodes), organogenesis (downward growth of hair placodes), and cytodifferentiation (morphogenesis of HFs). The dermis provides the initiation signal for HF development 16. In the embryonic stage, epidermal stem cells rapidly proliferate and differentiate to form hair placodes under the influence of signaling pathways such as the Wnt and BMP pathways. Hair placodes are a series of regular plate-like structures, and their formation indicates the commencement of HF development. After the formation of hair placodes, information is transmitted between two cellular compartments, epithelial and mesenchymal cells, which promote cell proliferation in both compartments by upregulating cyclins and facilitating hair germ formation. In these basal layer cells, upregulated Wnt-signaling induces the hedgehog signaling pathway, stimulating the proliferation of daughter cells with low levels of Wnt-signaling-related proteins. This process leads to the formation of an ORS and a bulge, which contain a pool of stem cells. Under signal stimulation, such as through the Wnt pathway, the hair embryo further proliferates into hair buds. Concurrently, hair placodes release signaling molecules, such as FGF20, into dermal fibroblasts, stimulating them to move directionally toward hair placodes, where they proliferate and aggregate to form the dermal condensate. The dermal condensate layer is then wrapped by HF epidermal cells, forming dermal papillae. Hair placodes grow toward the dermis. A group of highly proliferating cells at the bottom of the hair bud differentiates into matrix cells, forming the IRS 6, 17. Melanocytes localized to the ridge and stromal sites secrete melanin to impart color to the HS 15. Eventually, HF structures such as the IRS, ORS, and HS are formed.

## 4. HF cycle

The morphology of mammalian HFs changes periodically, resulting in the division of HF growth into anagen, catagen, and telogen phases. Each phase is tightly regulated and is characterized by substantial changes in gene expression, cell proliferation, and cell differentiation. During the anagen phase, the HF produces a complete shaft from the top to the root. The anagen phase determines the length of hair and depends on the continued proliferation and differentiation of stromal cells at the base of the follicle. The portion below the follicle bulge grows and thickens, and the hair papilla enlarges and gradually moves away from the bulge, eventually becoming enveloped by hairballs extending into the adipose tissue 18. The fat layer also thickens during the anagen phase, synchronized with the HF cycle. Adipocytes support the growth of HFs by producing various adipogenoids, such as leptin and adiponectin, and growth factors, such as macrophage colony-stimulating factor 18-20. As the hairballs continue to expand and descend toward the muscle membrane in the subcutaneous tissue, the HFs stop descending and enter a phase of vigorous growth. The transition from the growth phase to the catagen phase is facilitated by several factors, including FGF5, epidermal growth factor, neurotrophic factors (e.g., brain-derived neurotrophic factor and p75 neurotrophic factor receptors), p53. and TGFβ family members (e.g., TGF- β1) 21. After a period of continuous growth, HFs enter the apoptosis-mediated catagen phase. During the catagen phase, HFs stop growing, the lower portion of each HF is completely degraded 22, stromal cells and keratinocytes undergo apoptosis, and a circular structure called the bulb forms at the bottom of the dermis. This bulb ascends to the never-circulating HF to connect the hair DP with the bulge. When the DP reaches cells around the residual HS, it disappears completely. 21, 23. After the catagen phase concludes and the telogen phase commences, HFs remain relatively quiescent, storing numerous pluripotent stem cells at the bulge site, which are ready to receive signals from the DP 24. During the telogen phase, the DP is adjacent to the bulge, interacting with each other. When the stem cell activation signal reaches a threshold, the HFs begin to move into the next growth phase 25. When the DP is missing, HF stem cells cannot receive a complete signal, leading to the failure of the HF cycle 26.

## 5. Mechanisms underlying HF regeneration

HF regeneration is based on complex signal interactions between the HF stem cell pool and the hair DP. Ectoderm stem cells serve as the primary cell source for the regeneration of HF. Mesenchymal hair papilla cells regulate this process by secreting signaling molecules. Taylor et al. 27 used bromodeoxyuridine and 3H-thymidine double labeling to demonstrate the presence of numerous stem cells at the site of the follicle bulge. Stem cells at the bulge site work synergistically with the DP to maintain the self-renewal and periodic growth of HF. HF stem cells typically remain quiescent and exhibit slow periodic activity. However, when stimulated by damage or growth signals, they can proliferate rapidly, producing numerous transit amplifying cells and postmitotic differentiating cells, which play essential roles in skin damage repair and HF reconstruction 28, 29. A study in which HFs were cultured during the growth phase in four segments demonstrated that 95% of all clonal-forming cells originated from HF fragments in the bulge area 30, and a small number of cells were derived from the HF fragment of the hair bulb. HF stem cells mainly include ectoderm-derived hair keratinocyte stem cells, mesoderm-derived HF mesenchymal stem cells (MSCs), and neural crest-derived melanocyte stem cells 31. Under certain conditions, these cells can be induced to differentiate into adipose tissues, bone, and various other tissues. Dermal papilla cells (DPCs), which belong to a group of mesenchymal cells in the HF structure, are characterized by marked heterogeneity and adult stem cell properties**.** They have been confirmed to be a type of MSCs. DPCs are dermis-derived cells located at the base of HF and play pivotal roles in HF morphogenesis, HF regeneration, HF reconstruction, hair growth signal transmissions, and HF cycle regulation 32, 33. During the morphogenesis of HF, dermal fibroblasts receive signal stimuli from hair placode cells, which cause them to agglutinate and eventually develop into DPCs 34.

## 6. Current hair loss situation

Hair loss is a common and treatable condition that has evolved into a chronic problem, affecting a substantial portion of the population. An Israeli skin clinic reported that hair loss-related visits increased from 1.24% in 2010 to 9.44% in 2020. Furthermore, the highest increase was noted in the prevalence of androgenetic alopecia (AGA), from 17% in 2010 to 32% in 2020 35. Safavi et al. 36 indicated that the estimated incidence of AA was 20.2 per 100,000 person-years from 1975 to 1989. Furthermore, Mirzoyev et al. 37 reported that the estimated incidence of AA was 20.9 per 100,000 person-years from 1990 to 2009. Three epidemiological studies conducted in the United States have revealed a consistent increase, showing that the incidence of alopecia areata (AA) in the United States is increasing year by year over the years. Arash et al. 38 demonstrated that the estimated incidence of AA ranged from 91.46 per 100,000 person-years in 2016 to 92.90 per 100,000 person-years in 2019. In addition, hospital-based studies conducted in various countries, including India, Singapore, and Mexico 39-41, have reported that the incidence of AA ranges between 0.57% and 3.8% 42. Paige et al. 43 found that the prevalence of hair loss among pediatric patients (mean age, 9 years) had doubled in the last decade, indicating a trend of hair loss in younger individuals. Hair loss not only affects aesthetics but also imposes a psychological burden on individuals. Moreover, it negatively affects personal image, mental health, quality of life, and even social competitiveness. In response to the challenges posed by baldness, researchers are actively seeking solutions for hair loss 44. Recently, the demand for hair loss treatments has continually increased, reducing the incidence of mental and physical health disorders and the financial burden associated therewith 44. However, very few drug regimens have been approved by regulatory agencies for clinical use; these drugs include mainly corticosteroids, minoxidil, and 5-alpha-reductase inhibitors (finasteride and dutasteride) 45, 46. Although these medications have demonstrated effectiveness, their outcomes do not meet the needs of individuals with hair loss. Therefore, exploring new treatment approaches is imperative. The underlying causes of many types of hair loss involve the apoptosis and dysfunction of HF stem cells due to excessive hormone secretion, immune imbalances, and inflammatory response 25, 26, 47, 48. Addressing hair loss mainly entails inhibiting the apoptosis of HF stem cells, maintaining the homeostasis of HF stem cells, and ensuring the normal functioning of the HF of the hair follicle cycle.

## 7. Causes of hair loss

Common types of hair loss include AGA, AA, and telogen effluvium (TE). Among these types, AGA—also known as premature baldness, male baldness, and seborrheic alopecia—is the most prevalent. Androgenic baldness is a polygenic genetic disorder caused by androgens 49. HFs on the top of the head become more sensitive to androgens, leading to gradual HF atrophy, a shortened growth phase, a prolonged telogen phase, and eventual HF miniaturization, resulting in baldness 45. Typical symptoms include hair thinning starting from the frontal corners on both sides of the forehead, reduced hair density, and a receding hairline that gradually extends to the top of the head 50.

AA is a psychogenic-dominated, autoimmune-related hair loss disease characterized by a sudden onset of localized patchy alopecia. It is a complex disease caused by the interaction between genetic and environmental factors and can occur in any region of the body. The affected skin appears smooth, with no inflammation, scaling, or scarring. AA can occur at any age but is more common in young and middle-aged individuals, demonstrating no substantial sex differences 51. Typical symptoms include the sudden appearance of round or oval areas of hair loss with well-defined boundaries and varying numbers 52.

TE results from the disruption of the HF cycle, causing a large number of hairs in the anagen phase to simultaneously enter the telogen phase. This condition is characterized by a shortened anagen phase, a prolonged telogen phase, and the simultaneous shedding of the HS during the quiescent phase, resulting in the occurrence of telogen effluvium 53. The amount of hair loss is proportional to the number of HFs simultaneously entering the telogen phase; this condition is known as acute telogen phase alopecia. Under normal circumstances, the human scalp contains approximately 100,000 HFs, with 90%-95% in the anagen phase and 5%-10% in the telogen phase, cycling continuously. However, because the cycle of each HF is not synchronized, the daily hair loss in normal individuals typically remains under 100 54. Factors such as malnutrition, micronutrient deficiencies, chronic illnesses, emergencies, and mental stress can affect the growth cycle of HFs. If the HF cycle is disrupted, it can result in increased hair loss.

## 8. Conventional treatments for hair loss

Hair loss treatment is a crucial aspect of clinical dermatology. In recent years, many treatments have been proven to promote hair regrowth. Traditional drug and surgical interventions continue to play crucial roles in addressing hair loss.

### 8.1 Drug therapy

To date, minoxidil and finasteride are the main drugs approved by the US Food and Drug Administration (FDA) for the treatment of AGA. Minoxidil, which was originally used to treat hypertensive disorders, was later discovered to effectively control the life cycle of HFs; thus, it was established as the primary treatment for hair loss in men and women 55. Minoxidil is a potassium channel opener, exerting a vasodilating effect that extends the duration of the anagen phase and induces angiogenesis around the HFs 56. In 1999, Vera et al. 57 assessed the efficacies of 5% and 2% topical minoxidil against AGA; they observed that topical minoxidil promoted hair growth and delayed hair loss within 96 weeks. Recent studies have also highlighted the effectiveness of minoxidil in combination with other drug treatments for hair loss 55, 58.

Finasteride is another preferred drug for the treatment of AGA. Androgens affect the development of HFs, and androgenic metabolic changes are closely related to the occurrence of AGA 59. Testosterone is the main androgen in men; by contrast, the most crucial androgen in women is the weakly active form called androstenedione, which is secreted by the adrenal glands and ovaries 60, 61. Testosterone and androstenedione are metabolized and converted by 5-alpha reductase. Finasteride is a specific inhibitor of type II 5-alpha reductase, which can irreversibly bind to 5-alpha reductase to treat AGA 62. Piraccini et al. 59 evaluated the efficacy and safety of a topical finasteride spray in the treatment of AGA in men and reported that, compared with placebo, topical finasteride use significantly increased hair number.

Avodart is approximately thrice more potent than finasteride in inhibiting type II 5-alpha reductase 63. Olsen et al. reported that dutasteride increased the number of hairs in the target area in a dose-dependent manner and was more effective than finasteride at 12 and 24 weeks 64. Dutasteride is proposed as an effective therapy for frontal fibrosing alopecia 65 and is thus used as a new drug for AGA.

### 8.2 Surgical treatment

Hair transplantation involves the transplantation of hair extracted from a donor site into the bald area of the scalp (frontal lobe area/vertex). The preferred donor site is typically the occipital region of the scalp because of its resistance to androgens. When occipital hair is insufficient, hair can be harvested from areas such as the beard and chest 66. Follicular unit transplantation is the gold standard for hair transplantation 67. Two methods are used for graft harvesting: the strip method and follicular unit extraction. In the strip method, a section of the scalp is harvested from the occipital region, and individual follicles are dissected and then transplanted into a gap made in the recipient site. During the first few days to weeks after transplantation, some transplanted hair may fall out due to telogen shedding or transplant failure. Results typically become visible after at least 3 months, which is the time required for the transplanted hair to enter the anagen phase 67. Hair transplantation can be combined with other treatments such as oral finasteride, topical minoxidil, and PRP to improve outcomes 68. However, the essence of hair transplantation lies in the redistribution of self-dominant resources, and the low availability of donor sites limits the treatment satisfaction of hair transplant recipients 69.

## 9. New treatment strategies for hair loss

### 9.1 Physiotherapy

Scalp microneedle therapy, also known as micro needling or derma rolling for the scalp, is a non-surgical cosmetic procedure used to improve hair growth and overall scalp health. This procedure employs a device known as a microneedle roller, or derma roller. equipped with tiny needles on its surface. During treatment, a practitioner or an individual gently rolls the microneedle roller over the scalp. This action leads to the activation of genes associated with hair growth, the release of growth factors (e.g., platelet-derived growth factor), and the activation of stem cells with bulging hairs 70. Scalp microneedling is frequently used in combination with PRP to enhance drug absorption by creating microchannels. Kang et al. 71 also pointed out that micro needle (MN) and nanoparticles (NP) transport systems are emerging treatments for hair loss. Kim et al. 72 demonstrated that repeated microneedling induces hair growth. Possible mechanisms underlying the efficacy of microneedling against AGA include inducing the overexpression of hair growth-related genes (e.g., vascular endothelial growth factor, β-catenin, Wnt3a, and Wnt10b), regulation the hair cycle, stimulating DP, and promoting hair growth 73. The wound healing microenvironment created after microneedling treatment activates stem cells in the HF area, facilitating the delivery of drugs and increasing the absorption of various compounds in the skin 72. This method has been observed to be particularly effective when used in combination with minoxidil therapy or topical steroids. In general, microneedling aids the penetration of drugs such as minoxidil, topical steroids, and PRP. The ease of use, cost-effectiveness, and safety of microneedling make it a potentially attractive therapy for the treatment of hair loss 74.

Fractional radiofrequency (FRF) therapy involves the use of specialized equipment and needles to accurately deliver ultra-high-frequency radio waves to targeted tissues for the treatment of diseases. Verner and Lotti evaluated the efficacy of FRF in stimulating hair growth in 25 patients who completed 31 treatment sessions (between-treatment interval: 2 weeks). The researchers reported that hair density and HS increased by 6.18% and 39%, respectively, and that the treatment outcomes were tolerated well 75. Alsalhi et al. 76 demonstrated that FRF can enhance the delivery of topical minoxidil to the scalp. FRF not only reduces hair loss but also increases hair quantity and HS thickness. Another study indicated that FRF effectively reduced hair loss and stimulated hair growth in patients with AGA. A total of 25 patients completed 10 FRF sessions (administered every 2 weeks) and were followed up 2 months after the last session. The patients exhibited a 31.6% increase in hair counts and an 18% increase in HS thickness 77. Both microneedling and FRF are physical methods that can be combined with drugs to treat hair loss by promoting drug absorption and delivery.

### 9.2 Light therapy

#### 9.2.1 LLLT

LLLT, also known as photo biomodulation therapy, cold laser therapy, or red light therapy, is a non-invasive medical or cosmetic treatment that employs low-level lasers or light-emitting diodes to stimulate cellular function and promote tissue healing and regeneration. LLLT devices emit low-intensity laser or light-emitting diode light at specific wavelengths, typically in the red or near-infrared spectrum. These wavelengths range from 600 to 1000nm. LLLT operates through photo bioregulation, where red light triggers the release of nitric oxide from cytochrome C oxidase 78. This event leads to the production of additional adenosine triphosphate, and reactive oxygen species are produced by the redox reaction of cytochrome C oxidase, which activates DNA transcription factors. These transcription factors direct protein synthesis that is integral to cell proliferation, migration, and adhesion. LLLT enhances levels of cytokines, growth factors, inflammatory mediators, and tissue oxygenation 79. LLLT stimulates the generation of anti-inflammatory cytokines and antioxidants that accelerate the mitosis of keratinocytes and fibroblasts, ultimately promoting hair growth 80. Gentile et al. 81 conducted a randomized controlled trial comparing the effectiveness of LLLT in patients with male pattern hair loss (MPHL) and those with female pattern hair loss (FPHL) against a control group and reported that LLLT is useful for hair loss treatment. In a case series involving 20 patients with AGA, after 16 weeks of follow-up, hair density increased by 12 ± 2 hairs/cm^2^ 82, 83.

#### 9.2.2 Excimer lamp

Ultraviolet B light (290 to 320 nm) is widely used for treating AA 84, which is a T-cell autoimmune disorder. A 308-nm excimer laser has been reported to induce T-cell apoptosis in vitro 85. Zakaria et al. 86 conducted a blinded controlled clinical trial by using a 308-nm excimer laser for AA treatment; their findings indicated that all patients experienced ≥16% improvements in plaque after direct beam photography treatment 86.

#### 9.2.3 CO_2_ laser

The mechanism underlying CO_2_ fractional laser treatment for hair loss may involve the upregulation of Wnt β-catenin, which also enhances the delivery of traditional drugs, thereby promoting hair growth 87. Studies have observed a significant increase in hair density and hair diameter after 4 months of a low-energy, high-density regimen administered every 2 weeks (for a total of six sessions), indicating that CO_2_ laser therapy leads to positive outcomes within a relatively short period of the time 88. The efficacy of CO_2_ lasers is closely related to their energy parameters, which are often adjusted according to the patient's hair density or erythema area 89.

### 9.3 Regenerative medicine -based therapy

#### 9.3.1 PRP therapy

PRP is an autologous blood preparation characterized by a considerably higher number of platelets than that noted in whole blood 90. Upon activation, PRP releases a lysate rich in numerous growth factors, which play pivotal roles in promoting cell proliferation and tissue regeneration. The use of autologous PRP does not cause immune reactions, thereby broadening its applicability for various dermatological purposes, including wound healing, facial rejuvenation, scar repair, and hair growth 91, 92. Although the FDA has not approved the use of PRP for hair loss treatment, several studies have demonstrated its efficacy against AGA 93-95. Anitua et al. 96 evaluated the use of autologous PRP in 19 patients with AGA and observed significant increases in average hair density, hair diameter, and hair thickness at the 1-year follow-up. Alves and Grimalt 97 conducted a randomized, double-blind study involving 25 patients and noted that average hair count and hair density increased in PRP-treated areas 3 and 6 months after treatment. In another randomized, double-blind trial, Gentile et al. 98, 99 obtained the same results 3 months after PRP injection. These studies confirm the therapeutic advantages of PRP. Currently, PRP treatment for hair loss predominantly involves scalp injections, which can cause local infections, pain, bleeding, and uneven administration. Furthermore, multiple scalp injections can induce anxiety and fear in some patients 100-102.

#### 9.3.2 Stem cell therapy

Stem cells exhibit self-renewal, migration, anti-inflammatory, and immunomodulatory functions, which are essential for the repair and recovery of damaged tissues or organs 103-105.

Human DPCs (hDPCs) are specialized dermal cells located at the base of HFs and are surrounded by dermal sheath cells (DSCs). Both hDPCs and DSCs are necessary for new hair development and hair renewal. Notably, hDPCs can activate HFSCs and substantially influence HF circulation, thereby enabling HF regeneration. In transplant surgery, the efficiency of HF formation is related to the ability of DPCs to induce HF regeneration 106. In skin reconstruction and wound healing, DPCs not only retain the ability to form new DPCs but also promote the growth of DSCs and non-follicle-associated fibroblasts 107.Yamao et al. 108 co-transplanted highly passaged DPCs with cultured DSCs onto the dorsal side of nude mice. The researchers found that DSCs are involved in the induction of hair formation and that dermal sheath formation is key to the normal development of the HS. Ji et al. 5 investigated the HF regeneration mechanisms of DPCs and induced HF formation upon implantation in the hairless skin animals; their finding underscored that, when in contact with the epithelium, DPCs can generate new HFs. Tsuboi et al. 109 conducted a study involving 50 patients with MPHL who received a single injection of autologous DSC. The patient and 15 female pattern alopecia (FPHL)patients were randomized in a study, the results show, and the patient and 15 patients with female pattern alopecia (FPHL) who received a single injection of autologous DSCs. A substantial increase was noted in the number of DSC injection sites compared with the effects of a placebo intervention. Both men and women exhibited similar improvements. but neither sex had any severe adverse events 109. In the beauty industry, the transplantation of dermal fibroblasts into the scalps of patients with hair loss has been approved for regenerative product implementation 110.

MSCs are pluripotent stem cells with differentiation potential. Because of their crucial roles in enhancing the regenerative potential of various tissues and their secretion of various cytokines, nerve growth factors, and glial neurotrophic factors, MSCs have become integral to regenerative medicine 111, 112. Adult tissue sources of MSCs with self-renewal and multidirectional differentiation potential include HF-MSCs, human umbilical cord MSCs (hUC-MSCs), adipose MSCs (AD-MSCs), bone marrow MSCs (BM-MSCs), and mesenchymal-induced pluripotent stem cells (iPSC-MSCs). MSCs can regenerate HFs and other organs (e.g., sebaceous glands) in the skin through various mechanisms, including the reversal of pathological mechanisms and the formation of new HFs through organoid systems 113.

HF-MSCs possess several advantages over other adult tissue-derived stem cells. such as plentiful sources, easy accessibility, low immunogenicity, and no age restrictions, HF-MSCs play significant roles in HF development, the HF cycle, and HF regeneration. Kevin et al. 114 reported that HF-MSCs can promote chronic wound healing. Deng et al. 115 demonstrated that HF-MSCs reduced hair loss, alleviated inflammation around HFs, and increased follicle counts during the anagen phase in AA mice. Gentile et al. 116 117 reported that an RCT study treated with HF-MSCs, which showed a significant increase in hair follicle density and number compared to the control group, demonstrating the long-term therapeutic advantages of HF-MSCs.

hUC-MSCs are useful for tissue repair and regeneration. Their ethical advantage, painless retrieval from discarded umbilical cords, and reduced risk of immune rejection make hUC-MSCs more beneficial than other MSCs 118-120. Ko et al. 121 reported the crucial role of MSCs in wound healing. Exosomes from hUC-MSC can accelerate skin regeneration and wound healing 122. Cord mesenchymal stromal cell-derived exosomes can also rescue the loss of outer hair cells 123. Ahn et al. 124 successfully treated AA and generalized alopecia by using hUC-MSCs without administering immunosuppressive medications. These results indicate that hUC-MSC transplantation is safe and effective for the treatment of AA, with no hair loss during treatment or follow-up, no recurrence of hair loss or other side effects, and no immune rejection.

MSCs obtained from the adipose tissue include freshly derived primary pluripotent MSCs, known as adipogenic stromal vascular cells or adipogenic regenerative cells, as well as isolated and cultured pure MSCs (AD-MSCs). Saczonek et al. 113 indicated that AD-MSCs are necessary for the activation of epidermal stem cells in the skin and the normal cell growth of HFs. AD-MSCs secrete growth factors that play key roles in the activation of epidermal stem cells and hDPCs. These growth factors include vascular endothelial growth factor, which regulates hair growth and HF size by stimulating angiogenesis; hepatocyte growth factor, which is involved in determining the length of various stages of the hair cycle; platelet-derived growth factor, which induces and maintains the growth phase; and insulin-like growth factor-1, which controls the cycle of hair growth and hair cell differentiation 125-127. Adipose tissue is essential for the extension of the anagen phase. During the transition of hair from the telogen to anagen phases, adipose progenitor cells are activated to proliferate and form mature adipocytes around regenerating HFs 128. Zanzottera et al. 129 applied autologous AD-MSCs to the scalp of three patients and observed rapid wound healing, and improved hair growth, and a shortened telogen phase after 2 months of treatment. In their review, Yusuke et al. 75 indicated that AD-MSCs can improve the quality of aging skin and that the clinical use of these MSCs is allowed in dermatology and aesthetic surgery clinics in Japan, which highlights the safety and efficacy of adipose tissue-derived regenerative cells in the treatment of AA. According to Perez et al. 130, 19 out of 20 patients exhibited significant increases in hair diameter and hair density 3- 6 months after treatment 130. A study including 50 patients with MPHL or FPHL investigated the effects of adipose-derived regenerative cells on hair growth by combining these cells with adipose tissue during transplantation. The results revealed a 7% increase in the average hair volume, compared with a 5% increase in patients receiving nonadjuvant adipose tissue treatment alone 131.

Elmaadawi et al. 132 examined the safety and efficacy of autologous BM-MSCs in combination with autologous follicular stem cells (FSCs) in 20 patients with AA and 20 patients with AGA. Each patient received an intradermal dose of BM-MSCs or FSCs, and the effects were examined after 6 months through immunostaining and digital dermoscopy. The results demonstrated that both autologous BM-MSCs and FSCs were safe, well-tolerated, and effective in the treatment of drug-resistant AA and AGA. No side effects were noted. A significant improvement in hair growth was observed in all patients, with no significant between-group differences.

Induced pluripotent stem cells (iPSCs) have been used to generate various cell types, including HF cells 133. Subsequent studies have reported that HF-iPSCs can be used for hepatocyte regeneration in vitro 134. Recently, Abaci et al. 135 used three-dimensional (3D) printing technology to create micropores with adjustable extensions in plastic molds, mimicking the shape of HF. DPCs were added along with keratinocytes to form a 3D structure resembling HFs. After 3 weeks of culture, HF-like structures of cells expressing hair-specific markers and hair fibers were observed, confirming that functional HFs can be generated using biomimetic developmental methods. In addition, human keratinocytes,when embedded in the Matrigel matrix and combined with nylon guidewires as scaffolds, can assemble human iPSC (hiPSC)-derived DPC aggregates into a 3D structure to reconstruct HFs 136. Tsuji et al. 137 successfully bioengineered a 3D integumentary organ system comprising the skin, HF, sebaceous glands, and subcutaneous adipose tissue from mouse iPSCs (miPSCs). The resulting HF contained mature HF cells and structures such as bulge stem cells, melanocytes, DP, and DS. The hiPSCs have unlimited self-renewal capacity, which helps minimize the problem of cell shortages, and hiPSC-derived dermal and epidermal cells can induce hair genesis 138-140. However, the disadvantages of hiPSC-induced hair regrowth cannot be ignored. Differentiation of hiPSCs into hair dermal cells or follicular epidermal cells is more time-consuming than the isolation and expansion of these cells from HFs. In addition, when using any hiPSC-derived product for cell-based therapy, the possibility of tumor formation in hiPSCs should be considered 141.

### 9.4 Non-medical approaches

Dietary supplementation with natural plant extracts, controlled shampoo use, and lifestyle changes are widely accepted nonmedical strategies for preventing hair loss. For a long time, the consumption of natural plants and their extracts has been recognized as a safe approach with minimal side effects for preventing hair loss. Major plants known for hair loss prevention include ginseng and Polygonum multiflorum 142. Ginsenosides are among the main extracts of ginseng. Ginsenosides are classified into ternary diols (Rb1, Rb2, Rb3, Rg3, Rc, Rd, and Rh2), ternary alcohols (Re, Rf, Rg1, Rg2, and Rh1), and oleanolic acids. Ginsenoside Rb1 is the main bioactive component of ginseng and the most abundant ginsenoside in this plant 143. P. multiflorum is an oriental traditional medicinal plant. It is the tuber root of P. multiflorum Thunb (Polygonum family). The main extracts of P. multiflorum are 2,3,5,40-tetrahydroxystilbene-2-O-D-glucoside and emodin 144. In brief, the beneficial effects of herbs and their bioactive compounds and their potential mechanisms of action include the regulation of growth factors and cytokines, modulation of the Wnt/β-catenin pathway, inhibition of 5-alpha-reductase, involvement in sonic hedgehog signaling, and control of apoptosis and cell cycle progression. Park and Lee 142 comprehensively explored this subject in their review. Although phytochemicals have potential for treating alopecia, they have several limitations, including the limited availability of clinical evidence, varying levels of effectiveness, and slow appearance of results. In addition, phytochemical treatments are less effective for individuals with more severe hair loss 145. Controlled shampoo use and lifestyle modifications are more direct approaches to the prevention and treatment of hair loss than phytochemical use. Shampoo effectively removes dirt, excess oils, sweat, and environmental pollutants from the hair and scalp, promoting cleanliness and scalp health. Regular use of shampoo contributes to maintaining good scalp hygiene and reducing the risk of scalp conditions such as dandruff and fungal infections. However, excessive shampooing can strip the scalp of its natural oils, potentially leading to dryness, itchiness, and oil overproduction ("rebound effect"). In addition, some shampoos contain sodium lauryl sulfate, parabens, formaldehyde, and alcohol; these chemicals have been widely reported to damage the scalp and HFs 146. Even when using-silicone free shampoos, frequent washing over time may cause hair to become dependent on them because the scalp adjusts its oil production on the basis of the frequency of shampooing. This causes difficulty in reducing the frequency of shampooing. Therefore, selecting the right shampoo for your hair type and needs and monitoring usage frequency are essential. Finally, regardless of the nonmedical strategy used for treating hair loss, lifestyle changes are consistently advocated. A healthy lifestyle includes adequate hydration, a proper diet, and regular and adequate sleep. A balanced, nutrient-rich diet and proper hydration are essential for healthy hair growth. Hair is mainly made up of a protein called keratin. Thus, consuming adequate protein promotes hair growth. Lack of water can lead to dry hair. In addition, vitamin Bs, vitamin D, iron, zinc, and n-3 polyunsaturated fatty acids are essential for hair health. Foods such as lean meat, fish, eggs, nuts, fruits, vegetables, and whole grains should be included in the diet 147. Furthermore, stress management techniques such as meditation, yoga, and deep breathing exercises can help prevent hair loss and improve overall physical health. In summary, nonmedical treatment strategies generally have fewer side effects than pharmacologic therapies or surgical interventions. Moreover, nonmedical approaches are cost-effective, which makes them accessible to a wider population. However, the long-term cost of the sustained use of these nonmedical approaches should be considered. Non-medical treatments may not lead to satisfactory outcomes in individuals with severe hair loss.

## 10. Conventional methods versus new strategies

Although conventional treatments for hair loss are highly effective, they are associated with considerable challenges that limit their routine clinical use. Pharmacological treatments for hair loss can lead to various side effects. For example, minoxidil mainly causes hirsutism and cardiovascular symptoms/signs in a dose-dependent manner, and oral finasteride and dutasteride are associated with sexual dysfunction and neuropsychiatric side effects 55. Mouna et al. 148 reported cases of minoxidil-induced paresthesia, which may be due to irritant dermatitis caused by topical minoxidil. Studies have reported an increase in the number of hairs in women who continue to use minoxidil solution topically, but this effect disappears once the drug is discontinued 149. Adverse events related to finasteride are primarily associated with the disruption of the balance between estrogen and androgens due to 5-alpha reductase inhibitors. This imbalance can increase the risk of breast cancer and result in other adverse effects, such as reduced sexual function, testicular pain, and breast tenderness 59. The key surgical treatment for hair loss is hair transplantation, which involves the redistribution of self-dominant resources; however, the limited availability of donor sites reduces the treatment satisfaction of hair transplant recipients 69.

### 10.1 Minoxidil versus finasteride versus dutasteride

Minoxidil, finasteride, and dutasteride are the most common drugs used in hair loss therapy. Khandpur et al. 150 compared oral minoxidil, finasteride, and dutasteride in terms of their efficacy against AGA. The researchers revealed the following descending order of efficacy: avodart at 0.5 mg/day > finasteride at 5 mg/day > minoxidil at 5 mg/day > finasteride at 1 mg/day > minoxidil at 0.25 mg/day 150.

### 10.2 Microneedling versus minoxidil and finasteride

Lin et al. 151 examined the effects of 5% minoxidil and found that the mean improvement in total hair density from baseline to week 24 was 18.8/cm2 in patients who had received topical application alone and 38.3/cm2 in those who had received electrodynamic microneedling therapy plus topical 5% minoxidil.

### 10.3 LLLT versus minoxidil and finasteride

One study compared oral finasteride, topical minoxidil, and LLLT in terms of their efficacy against hair loss. The findings revealed that treatment with 1 mg of finasteride for 6 and 12 months resulted in 7.3% and 8.99% increases in the total hair count, respectively 152. Treatment with 2% and 5% minoxidil for 48 weeks increased the total hair count by 8.84% and 12.3%, respectively 153. After 26-week-long LLLT with 9- and 12-beam laser combs, the total hair count increased by 12.79% and 16.96%, respectively 80. Thus, the effectiveness of LLLT may be comparable to that of conventional hair loss treatments 81.

### 10.4 PRP versus finasteride

Gentile et al. 154 reported that posttreatment hair density was significantly higher in the PRP group than in the control group. After 12 weeks of treatment, the HF density increased by 31% ± 2% in the PRP group and by <1% in the control group. Nestle et al. 155 examined 212 patients with AGA who received 1 mg of finasteride daily. After 48 weeks of treatment, the HF density increased by 26% ± 3.1% in the finasteride group. The efficacy of PRP in promoting HF density was significantly stronger than that of finasteride.

### 10.5 PRP versus HFSCs

Gentile et al. 116 reported that after 23 weeks of treatment with HFSCs, HF density increased by 29% ± 5% hairs/cm^2^ compared with the density in the control group. After 23 weeks of PRP treatment, HF density increased by 28% ± 2% hairs/cm2 compared with that in the control group 154. These studies demonstrate that PRP and HF-MSCs are equally effective in treating hair loss 156.

## 11. Conclusions and perspectives

Hair loss is a difficult-to-treat condition, and treatment with FDA-approved drugs alone is often associated with substantial side effects and recurrence after discontinuation. Various strategies for treating hair loss are currently being explored. New strategies, including regenerative medicine-based therapy, remain attractive emerging options. Although new strategies for hair loss treatment are effective, they still have some limitations and uncertainties, and more clinical treatment cases are needed for validation. Our research group has also been committed to studying HF development and regeneration. Some of our findings are as follows; Ggene therapy can promote HF proliferation and regulate their cycle processes 157-159. Stem cell-derived exosomes or growth factors promote wound healing and HF proliferation 160, 161. HF regeneration can be induced through tissue engineering 162. HF-induced pluripotent stem cells can be induced through gene reprogramming 157. The HF microenvironment regulates the HF cycle process 163, 164. In the future,we will collaborate with other researchers to resolve the problem of hair loss and ensure sustainable clinical applications of effective therapeutic strategies.

### Author Contributions

D. L. wrote the draft of the manuscript. Q. X. and X.M. were involved in discussing, drafting, and editing the manuscript. All authors contributed to the article and approved the submitted version.

### Funding

This research was funded by National Natural Science Foundation of China grant numbers 82073581 and 82273673.

## Figures and Tables

**Figure 2:**
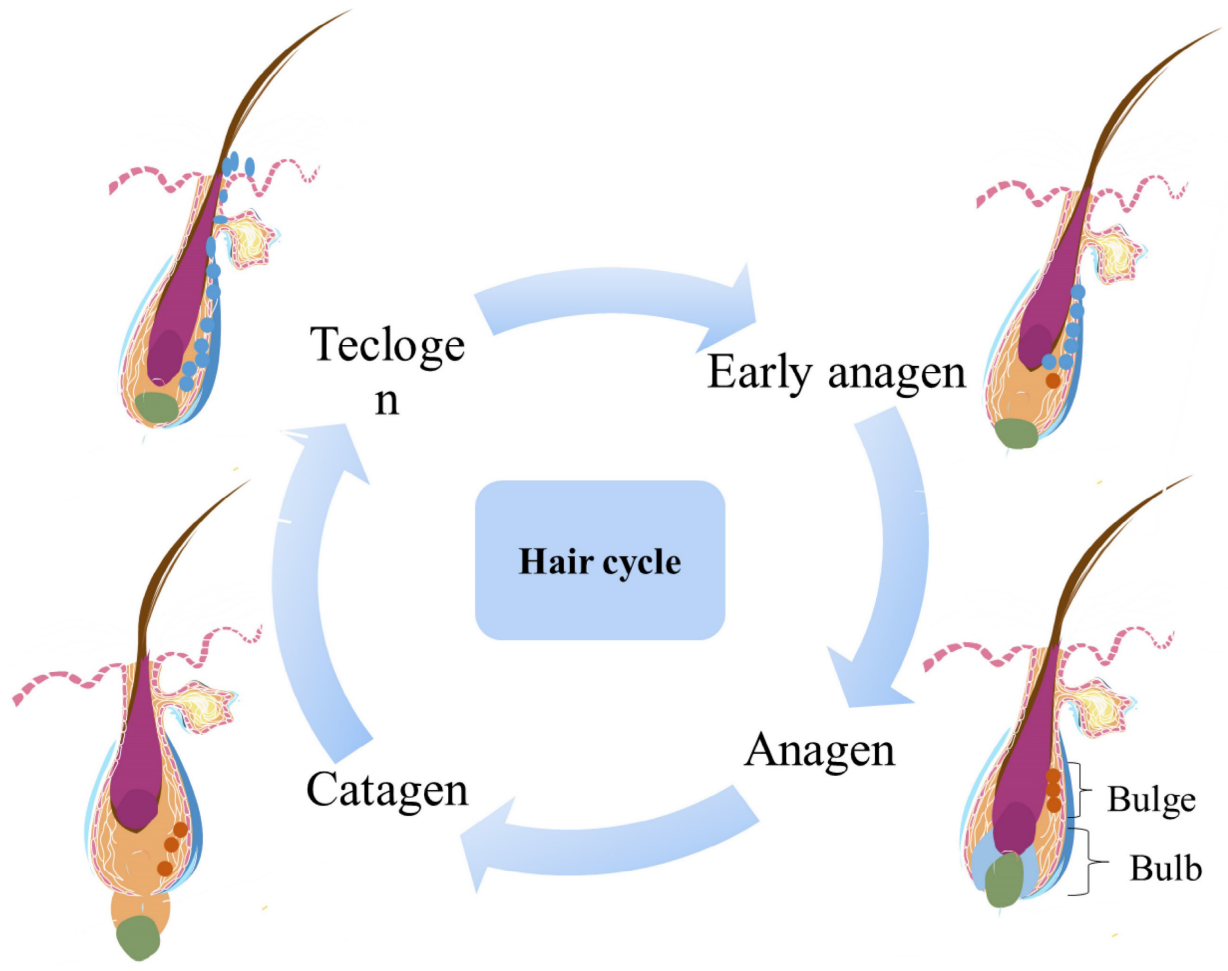
Hair follicle cycle.

**Figure 3:**
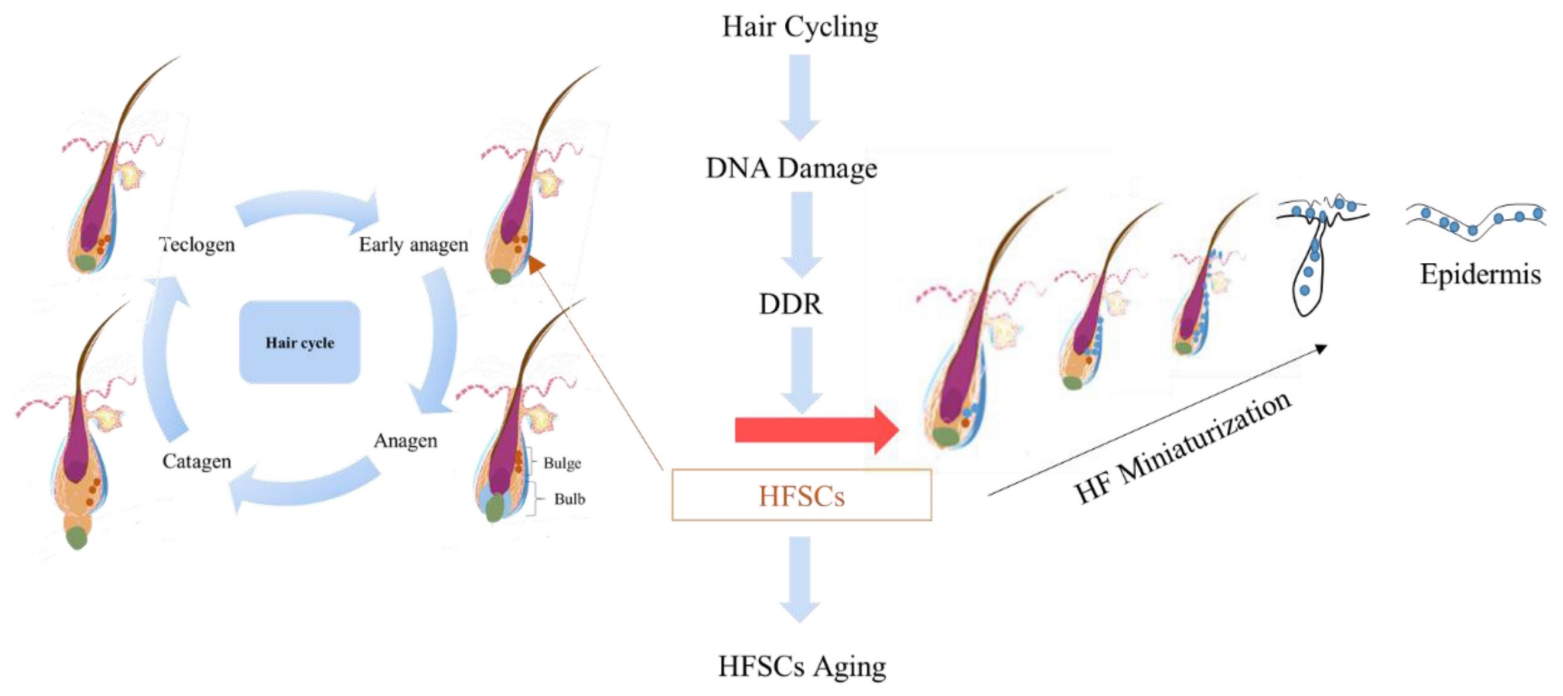
Mechanisms underlying the generation of hair follicles.

**Table 1:** Representative studies of traditional methods of hair loss treatment

Treatment	Conventional methods			
	Minoxidil	Finasteride	Dutasteride	Hair transplantation
**Types**	TE	AGA	FFA	AGA and FFA
**Patients**	58 patients	458 patients	224 patients	Four cases
**Dosage**	0.75-2.5 mg for 2.1 years	0.25% solution once daily (volume of 50-200 µL)	From 1-7 capsules (0.5 mg) per week	Artificial hair implantation
**Treatment results**	Hair density↑	Hair count↑	Distance of the eyes from the hairline↓	Hair count↑
**Adverse effects**	Headaches, hypertrichosis, andirritation	Sexual dysfunction, altered libido, gynecomastia, and mood changes	Sexual dysfunction, altered libido, gynecomastia, and mood changes	Pain, bleeding, edema, and pruritis
**References**	147	59	65	70

**Table 2:** Representative studies of new strategies of hair loss treatment.

Treatment	New strategies									
	Microneedling	FRF	LLLT	Excimer lamp	CO_2_ laser	PRP	DPC	UC-MSCs	AD-MSCs	BM-MSCs
**Types**	AGA and AA	AGA	AGA	AA	AGA	AGA	PHL	AA	AGA	AA and AGA
**Patients**	50 patients	25 patients	110 patients	105 patients	45 patients	25 patients	65 patients	3 patients	3 patients	40 patients
**Dosage**	Microneedling (needle length, 0.5 to 1.5 mm), 15 times a week	Ten treatment sessions at 2-week intervals	For 15 min, thrice a week for 26 weeks	308-nm excimer lamp	50-mm tip, 12-18 mJ/spot, 361 spots/cm^2^	Once a month for 3 months	7.1 × 10^7^ autologous DSCs	1 × 10^6^ cells/mL, twice a month for 12 months	Cell suspension, injected subcutaneously	1 × 10^4^ cells/mL, injected intradermally
**Treatment results**	Hair shaft thickness↑	Hair count↑ Hair shaft thickness↑	Hair density↑ Hair loss↓	Hair loss↓	Hair count↑ Hair thickness↑	Hair density↑	Hair density↑	Hair regrowth↑	Wounds↓ Hair regrowth↑	Hair regrowth↑
**Adverse effects**	Pain, bruising, and discomfort at site	Scalp pain and slight burning sensation	Scalp tenderness, paresthesia, and mild urticaria	Scalp pain and slight burning sensation	Scalp tenderness and paresthesia	Scalp pain and pinpoint	Scalp pain and pinpoint	Scalp pain and pinpoint	Scalp pain and pinpoint	Scalp pain and pinpoint
**References**	74	77	82	86	150	97	108	122	127	130
